# Risk and Protective Factors for Gambling Among Youth by origin: Findings from the three waves of cross-sectional Finnish School Health Promotion Study among 238,939 Students

**DOI:** 10.1007/s10899-024-10321-7

**Published:** 2024-07-29

**Authors:** Kirsimarja Raitasalo, Johanna Järvinen-Tassopoulos, Shadia Rask, Natalia Skogberg

**Affiliations:** 1 Finnish Institute for Health and Welfare, Helsinki, Finland; 2 University of Eastern Finland, Kuopio, Finland; 3 Faculty of Social Sciences, Centre for Research On Addiction, Control, and Governance, University of Helsinki, Helsinki, Finland

**Keywords:** Gambling, Adolescents, Immigrant background, Survey, Finland

## Abstract

Gambling is a public health problem that can cause many kinds of harm. The aim of this study was to examine youth gambling by origin, and the risk and protective factors associated with it. The data was drawn from the School Health Promotion Study (n = 238,939) conducted in Finland, representative of the 14 to 16-year-old Finnish schoolchildren. Cross-tabulations and multivariate logistic regression were used in assessing the association between origin and weekly gambling. Interaction terms of origin and background variables related to substance use, peer and family relations and leisure time were then calculated to assess inter-group differences. The study showed that foreign-born, migrant origin and youth from mixed families were more likely to gamble weekly compared to youth with Finnish-born parents. The likelihood of gambling was particularly high among foreign-born and migrant-origin youth. Weekly gambling was significantly more common among boys than girls in all studied youth groups, and it was particularly common among foreign-born boys compared to other groups. Substance use was associated with weekly gambling and even more so among foreign-born youth. There were also differences by origin in the strength of association between other background factors and weekly gambling. Foreign-born boys appear to be especially vulnerable to multiple health and social risks including gambling, making them a particularly important group for targeted preventive programs. Preventive efforts are needed to enhance public awareness, boost parental supervision, and limit gambling-related risks. Special attention is needed to prevent migrant-origin boys from developing problems with gambling.

## Introduction

Gambling is a public health problem that can cause social, economic, mental health and health-related harms to individuals and their significant others (Price et al., 2021). Persons at a higher risk of vulnerabilities, including youth and persons of migrant origin are disproportionally more likely to gamble than other population groups (Campisi et al., 2017; Raisamo et al., 2020). Early initiation to gambling, which may occur through one’s own gambling at a young age or exposure to parents’ gambling or problem gambling, has been shown to predispose individuals to problems later in life (Donati et al., 2021; Spångberg & Svensson, 2022). In addition to adverse social, economic and health impacts, problematic gambling among youth can have a negative impact on their development and school performance and may increase their risk of other addictions (Armitage, 2021).

Gambling has previously been reported to be more common among youth in Finland than in most other European countries. On average, 14 percent of European adolescents in 2019 had gambled within the preceding year, while in Finland the corresponding proportion was 24 percent (ESPAD Group, 2020). In most European countries including Finland, all types of gambling are prohibited for minors (Spångberg & Svensson, 2022). However, previous studies have shown that despite these age limits, European youth still gamble, both offline and online (Andrie et al., 2019; ESPAD Group, 2020; Molinaro et al., 2018). Youth are exposed to gambling in various ways, e.g., through marketing, but also while watching sports and playing online games featuring an in-game gambling option (Armitage, 2021; Thomas et al., 2023). Furthermore, many countries have a rather permissive gambling culture, as gambling is seen as a socially accepted form of entertainment (Derevensky et al., 2005).

Inequalities in health and wellbeing are found in the School Health Promotion Study with foreign-born youth faring worse than Finnish-born peers across various indicators of wellbeing. Key risk factors related to the family include parents’ prolonged unemployment, low education level, and financial problems, as well as parental mental health and other health problems, or being a single parent (Kääriälä et al., 2020). Individuals of migrant origin are at a greater risk of unemployment, more likely to be overqualified for their jobs, and over-represented in low-paid jobs and in-work poverty as compared to their native-born peers (e.g., Bárcena-Martín & Pérez-Moreno, 2017; Kaida, 2015; OECD, 2017). Overall, earnings and employment improve with time spent in the country, but the native-migrant gap often remains in Finland as well (OECD, 2018). Long-term poverty is known to be detrimental for various outcomes, and even across generations (Lesner, 2018).

Engagement in gambling activities in adolescence has been shown to be associated with the use of alcohol, tobacco, and illicit substances (Castrén et al., 2015, 2021). Peers also have an important role in and a strong influence on risky behaviors in adolescence, such as substance use and gambling (Delfabbro et al., 2016; Helmer et al., 2021; Kristjansson et al., 2013; Zhai et al., 2017). Furthermore, research on parenting styles has shown that consistency in parenting, parental monitoring and a positive relationship with parents are important factors that protect youth from various problematic behaviors, including gambling (Sobotková et al., 2013; Canale et al., 2016; Kveton & Jelınek, 2016; Molinaro et al., 2018; Kapetanovic et al., 2020; Pisarska & Ostaszewski, 2020). Individual behaviors such as going out in the evening also increased the likelihood of being a gambler (Molinaro et al., 2018). These risk and protective factors may be differently associated with gambling in different migrant-origin groups (Järvinen-Tassopoulos & Raitasalo, 2017).

While gender and age are the two socio-demographic factors that have been consistently shown to be associated with youth gambling and problem gambling, surprisingly little research has been carried out on the topic among migrant-origin youth. In countries where migration is a fairly recent phenomenon, the number of participants of migrant origin has been very small. Also, few studies have used data which distinguishes between so-called first- and second-generation migrants, and children born into mixed families (Campisi et al., 2017).

Despite the growing size of the migrant-origin population in Finland (458,000, 8.3% of the Finnish population at the end of 2021) (https://www.stat.fi.), only a few studies have reported differences in risk behaviors between native and migrant origin youth in the Finnish context (e.g., Matikka et al., 2014; Kataja et al., 2022). According to the Finnish School Health Promotion Study 2013, migrant-origin youth gambled (any kind of gambling product) more often than Finnish-origin youth. A gradient in gambling was reported according to origin. Foreign-born youth reported higher prevalence of gambling compared to youth of migrant origin born in Finland, youth born into mixed families and youth with Finnish-born parents. The probability of gambling was higher among foreign-born youth who have lived in Finland for less than 10 years. In all groups of origin, gambling was associated with substance use (Räsänen et al., 2016). Another study based on Finnish data from the European School Survey on Alcohol and Drugs Project (ESPAD) focusing on slot machine gambling among youth also included analyses by gender and found no differences between migrant and Finnish-origin boys, whereas gambling was significantly more common among migrant-origin girls compared to Finnish-origin girls (Järvinen-Tassopoulos & Raitasalo, 2017). Still, a comprehensive understanding of factors associated with gambling by origin is lacking.

This study is set in Finland, a Nordic country characterized by a generous welfare state, a relatively high level of income equality, and comparatively well-developed policies for migrant integration (Malmusi et al., 2010). The Finnish gambling system is based on a state monopoly, and since 2017, there has been only one state-owned gambling company (Veikkaus). Since 2011, the age limit of all types of gambling has been 18 years. A particular characteristic of Finland is the prevalence of electronic gambling machines in everyday locations, such as supermarkets, kiosks, and gas stations (Järvinen-Tassopoulos et al., 2021).

In this study, we are interested in whether there are differences in the probability of gambling among youth by origin. We also aim to shed light on different risk and protective factors associated with gambling among youth and whether these differ by origin. We expect to find variation in the prevalence of gambling across the studied groups, with gambling being more prevalent among migrant-origin than among Finnish-origin youth. We also expect to find gender differences, with gambling being more prevalent among boys than girls in all of the studied groups.

The research questions are:

1. Are there differences in the prevalence of weekly gambling between different migrant-origin and Finnish-origin youth groups?
2. How are other known risk and protective factors for gambling (substance use, sports activity, school orientation, general health, life satisfaction and peer- and family-related factors) associated with weekly gambling and do these possible associations differ by origin?

## Methods

### Sample

This study utilized data from the Finnish School Health Promotion Study (SHP) administered by the Finnish Institute for Health and Welfare from three cross-sectional data collection waves in 2017, 2019 and 2021 (THL, 2023). SHP gathers information on well-being, health, schoolwork, attendance and participation of children and adolescents and has been administered every second year since 2013. All students in Finland in 4th, 5th, 8th and 9th grades in primary education and 1st and 2nd year students in upper secondary schools and vocational schools are invited to participate in the study. Students responded anonymously, independently, and voluntarily during the school day under supervision. Data were collected with an online or a paper and pencil survey. The study was conducted according to the guidelines of the Declaration of Helsinki and was approved by the Ethics Board of the Finnish Institute for Health and Welfare.

The data used in this study consists of the answers of the 14 to 16-year-old students studying in the 8th and 9th grade in primary school from three consecutive data collection years: 2017 (*N* = 68 992, data coverage 64%), 2019 (*N* = 82 875, data coverage 75%) and 2021 (*N* = 87 072, data coverage 77%). These three data sets were pooled together to get more respondents in each origin-based subgroup of students.

### Measures

#### Outcome Variables

Weekly gambling was assessed with the question ‘How often do you gamble?’ (‘6–7 days a week’, ‘3–5 days a week’, ‘1–2 days a week’, ‘less often than once a week’, ‘less often than once a month’, ‘I have not gambled during the past year’. Those responding, ‘6–7 days a week’, ‘3–5 days a week’ or ‘1–2 days a week’, were defined as weekly gamblers.

#### Independent Variables

Origin was categorized based on respondents’ own and their parents’ country of birth: 1) respondent and both parents born in Finland (later in the text referred to as youth with Finnish-born parents); 2) respondent with one parent born abroad, other parent born in Finland (later in the text referred to as ‘youth from mixed families’); 3) respondent born in Finland with both parents foreign-born (later in the text referred to as ‘migrant-origin youth’), 4) respondent and both parents foreign-born (later in the text referred to as ‘foreign-born youth’).

Heavy episodic drinking (HED) during the past 30 days was measured by asking ‘How often do you consume alcohol until you are heavily drunk?’ (‘once a week or more often’, ‘about 1 to 2 times a month’, ‘less frequently’, ‘never’). The responses were collapsed into a dichotomous variable (0 = ‘no’, 1 = ‘once or more often’).

Daily smoking was assessed with the question ‘Which of the following responses best describe your current smoking habits?’ (‘I smoke once a day or more often’, ‘I smoke once a week or more often, but not every day’, ‘I smoke less often than once a week’, ‘I have quit smoking’.) Those who responded ‘I smoke once a day or more often’ were defined as ‘daily smokers’.

Lifetime drug use was measured using the following series of questions: ‘Have you ever tried or used…?’ a) marijuana or hashish (cannabis), b) ecstasy, c) amphetamines, d) subutex, e) heroin, f) cocaine, g) LSD, h) gamma or similar narcotic substances. (‘never’, ‘once’, ‘2–4 times’, ‘5 times or more’). Those who selected any other response than ‘never’ for any of these questions were defined as ‘lifetime drug users’.

Weekly sports activity was assessed with the question ‘How often do you exercise or participate in sports a) led by an instructor or b) on your own initiative in your leisure time?’ (‘almost daily’, ‘every week’, ‘every month’, ‘less frequently’, ‘never’). Those who responded ‘almost daily’ or ‘every week’ to either of these options were defined as ‘active in sports’.

Liking school was defined using the question ‘How do you like school at this moment? I like school… (‘very much’, ‘quite a lot’, ‘fairly little’, ‘not at all’). Those who responded ‘very much’ or ‘quite a lot’ were defined as ‘liking school’.

The students were asked ‘How satisfied are you with your life at the moment?’ (‘Very satisfied’, ‘Fairly satisfied’, ‘Neither satisfied nor dissatisfied’, ‘Fairly dissatisfied’, ‘Very dissatisfied’. Those who responded ‘Very satisfied’ or ‘Fairly satisfied’ were defined as being ‘satisfied with life’.

General health was assessed with the question ‘How is your health in general?’ (‘very good’, ‘fairly good’, ‘average’, ‘fairly or very bad’). Those who responded ‘very good’ or ‘fairly good’ were defined as ‘being in good health’.

Having close friends was defined using the question ‘At the moment, do you have a close friend with whom you can talk confidentially about almost everything concerning yourself?’ (‘I do not have any close friends’, ‘I have one close friend’, ‘I have two close friends’, ‘I have several close friends’). Those who selected any response other than ‘I do not have any close friends’ were defined as ‘having close friends’.

Dialogical connection with parents was assessed with the question ‘Can you talk about things that concern you with your parents?’ (‘Hardly ever’, ‘Occasionally’, ‘Fairly often’, ‘Often’). Those who selected any other response than ‘hardly ever’ were defined as ‘having a dialogical connection’.

The family’s financial situation was defined as ‘good’ if the student chose either of the two first response options to the question ‘How would you rate your family’s financial situation?’ (‘Very good’, ‘Fairly good’, ‘Moderate’, ‘Fairly poor’, ‘Very poor’).

Sex (boy/girl) was based on self-report.

The variables with their distributions used in the analysis are presented by study year in Table 1.

**Table 1:** Characteristics of the participants by data collection year

	%, 2017 (*N* = 68 992)	%, 2019 (*N* = 82 875)	%, 2021 (*N* = 87 072)
Weekly gambling	6.7%	4.4%	3.9%
Boy	48.1%	48.1%	47.7%
Girl	51.9%	51.9%	52.3%
Both parents Finnish-born	86.8%	86.8%	86.0%
Mixed families	7.4%	7.5%	7.6%
Migrant origin, born in Finland	2.0%	2.2%	2.5%
Foreign-born	3.9%	3.6%	3.9%
Monthly HED ^1^	9.7%	9.0%	8.6%
Daily smoking	6.4%	5.2%	5.0%
Lifetime drug use	7.4%	8.4%	7.7%
Active in sports	76.7%	71.9%	72.3%
Likes school	60.7%	60.8%	58.4%
Satisfied with life	71.9%	69.6%	75.8%
Good health	81.4%	79.2%	74.3%
Has close friends	91.8%	91.2%	90.8%
Can talk with parents	92.3%	93.6%	92.2%
Good financial situation	68.0%	74.7%	74.3%

^1^HED, heavy episodic drinking

### Statistical analysis

Cross-tabulations were used to study how weekly gambling as well as the selected background factors were distributed among youth by origin and gender. Multivariate logistic regression was used in assessing the association between origin and weekly gambling. All the background variables along with the year of study were added in the adjusted model to see whether these factors have any effect on the association between gambling and immigrant background status. Finally, separate models were conducted for youth by origin to see whether the risk and protective factors were similar or different in separate groups. Interaction terms of origin and each background variable were then calculated to assess the statistical significance of possible between-group differences. The SAS enterprise guide 8.3 was used in all the analysis (SAS/STAT®, 2011).

## Results

Table 2 shows that regardless of origin, boys gambled more than girls. Foreign-born youth gambled remarkably more often than their peers born in Finland. Similar patterns by origin were also observed with respect to all types of substance use (alcohol, tobacco, and illegal drugs). Compared to their peers, foreign-born youth were also less active in sports, perceived their health worse, had fewer close friends and were less likely to discuss their problems with their parents. On the other hand, foreign-born boys had higher life satisfaction than their peers, while the opposite was true among girls. Foreign-born boys perceived their family’s financial situation as good less often than boys in the other study groups, while the variation among girls was less consistent.

**Table 2:** Characteristics of the study population by origin and sex: results from 2017–2021

	Both parents Finnish-born		Mixed family		Migrant origin, born in Finland		Foreign-born	
	Boys, %	Girls, %	Boys, %	Girls, %	Boys, %	Girls, %	Boys, %	Girls, %
	*N* = 98876	*N* = 107854	*N* = 8080	*N* = 9777	*N* = 2445	*N* = 2856	*N* = 5165	*N* = 9051
Weekly gambling	7.0	0.8	9.0	1.6	14.9	3.4	39.0	13.1
Monthly HED^1^	8.6	8.2	9.9	9.9	10.8	5.4	30.3	14.1
Daily smoking	5.3	4.6	5.4	6.1	7.0	4.0	24.1	11.7
Lifetime drug use	7.4	5.8	12.1	10.1	14.7	8.8	36.9	17.5
Weekly sports activity	74.7	74.6	72.8	70.6	69.6	58.1	58.9	51.7
Likes school	61.0	59.2	61.0	56.2	67.0	63.1	54.1	59.8
Satisfied with life	60.6	83.4	64.2	84.8	56.2	75.6	62.9	76.4
Good health	85.9	72.3	82.0	66.3	84.0	69.3	75.2	66.7
Has close friends	90.3	93.6	87.7	92.0	85.9	89.8	72.6	82.8
Can talk with parents	96.0	91.2	93.8	89.0	91.2	88.0	76.2	83.7
Good financial situation	77.7	69.5	72.5	64.4	74.2	70.5	63.0	66.4

^1^HED, heavy episodic drinking

Table 3 shows a gradient in gambling by origin, with the lowest probability to gamble being among youth with Finnish-born parents and the highest among foreign-born youth. After adjusting for the year of data collection and all the independent variables, foreign-born youth had over five-fold probability for weekly gambling compared to youth with Finnish-born parents (AOR = 5.32, 95% Cl 4,91–5,76). Also, youth from mixed families had higher probability (AOR = 1.26, 95% Cl 1.16–1.38) as well as migrant-origin youth (AOR = 2.09, 95% Cl 1.82–2.40). All of the studied independent variables were statistically significantly associated with weekly gambling following adjustment, except for weekly sports activity.

**Table 3:** The associations of origin and risk and protective factors with weekly gambling, odds ratios (OR) with 95% confidence intervals (CI)

		OR (95% Cl)	AOR^1^ (95% Cl)
Immigrant background	Both parents Finnish-born Mixed family Migrant origin, born in Finland Foreign-born	1.00 1.31 (1.22–1.41) 2.44 (2.20–2.70) 9.89 (9.38–10.43)	1.00 1.26 (1.16–1.38) 2.09 (1.82–2.40) 5.32 (4.91–5.76)
Year	2017 (ref) 2019 2021	1.00 0.64 (0.61–0.67) 0.56 (0.53–0.59)	1.00 0.57 (0.54–0.61) 0.51 (0.48–0.54)
Sex	Boy (ref) Girl	1.00 0.14 (0.13–0.15)	1.00 0.13 (0.12–0.13)
Monthly HED	No (ref) Yes	1.00 7.85 (7.53–8.19)	1.00 3.29 (3.09–3.52)
Daily smoking	No (ref) Yes	1.00 7.98 (7.62–8.37)	1.00 2.00 (1.86–2.16)
Lifetime drug use	No (ref) Yes	1.00 8.27 (7.93–8.63)	1.00 2.44 (2.28–2.61)
Weekly sports activity	No (ref) Yes	1.00 0.66 (0.63–0.68)	1.00 1.03 (0.97–1.09)
Likes school	No (ref) Yes	1.00 0.44 (0.42–0.46)	1.00 0.58 (0.55–0.61)
Good health	No (ref) Yes	1.00 0.81 (0.78–0.85)	1.00 1.07 (1.00–1.14)
Has close friends	No (ref) Yes	1.00 0.34 (0.32–0.35)	1.00 0.66 (0.62–0.71)
Satisfied with life	No (ref) Yes	1.00 1.68 (1.61–1.78)	1.00 1.59 (1.51–1.68)
Can talk with parents	No (ref) Yes	1.00 3.68 (3.50–3.86)	1.00 0.44 (0.41–0.47)
Good financial situation	No (ref) Yes	1.00 0.79 (0.76–0.83)	1.00 1.06 (1.00–1.12)

^1^AOR, Adjusted odds ratio (adjusted for year of the data collection and all other independent variables)

Table 4 shows that girls had a statistically significantly lower probability for weekly gambling in all origin groups. The statistically significant interaction between sex and weekly gambling (*P* < 0.0001) showed a somewhat stronger association among youth with Finnish-born parents compared to the other study groups. Also, monthly HED and lifetime drug use were positively associated with gambling in all origin groups. The significant interaction of HED and lifetime drug use with weekly gambling showed a stronger association among foreign-born youth compared to youth with Finnish-born parents (HED *P* = 0.0058; drug use *P* < 0.0001). Daily smoking was positively associated with weekly gambling in all groups and no significant interaction was found.

**Table 4:** The association of risk and protective factors with weekly gambling by origin, odds ratios (OR) and their 95% confidence intervals (95% CI), adjusted for year of data collection and all other independent variables

		Both parents Finnish-born	Mixed family	Migrant origin, born in Finland	Foreign-born	Interaction Wald chisq, Pr(chisq)
		OR (95% CI)	OR (95% CI)	OR (95% CI)	OR (95% CI)	
Year	2017 (ref) 2019 2021	1.00 0.56 (0.53–0.60) 0.46 (0.43–0.50)	1.00 0.50 (0.41–0.62) 0.61 (0.50–0.74)	1.00 0.51 (0.35–0.74) 0.80 (0.58–1.11)	1.00 0.80 (0.66–0.98) 0.71 (0.59–0.86)	42.81 (*P* < .0001)
Sex	Boy (ref) Girl	1.00 0.10 (0.09–0.11)	1.00 0.15 (0.12–0.18)	1.00 0.20 (0.14–0.28)	1.00 0.23 (0.19–0.28)	70.70 (*P* < .0001)
Monthly HED	No (ref) Yes	1.00 3.16 (2.93–3.40)	1.00 2.92 (2.33–3.65)	1.00 4.46 (3.03–6.56)	1.00 4.45 (3.60–5.49)	12.54 (*P* = 0.0058)
Daily smoking	No (ref) Yes	1.00 1.86 (1.70–2.03)	1.00 2.37 (1.83–3.06)	1.00 1.81 (1.14–2.88)	1.00 2.35 (1.86–2.97)	5.902 (*P* = 0.1165)
Lifetime drug use	No (ref) Yes	1.00 2.17 (2.00–2.35)	1.00 2.71 (2.18–3.36)	1.00 3.46 (2.41–4.97)	1.00 3.62 (2.98–4.39)	28.78 (*P* < .0001)
Weekly sports activity	No (ref) Yes	1.00 1.10 (1.03–1.17)	1.00 1.04 (0.86–1.26)	1.00 0.83 (0.62–1.12)	1.00 0.83 (0.70–0.98)	11.87 (*P* = 0.0078)
Likes school	No (ref) Yes	1.00 0.57 (0.53–0.60)	1.00 0.70 (0.58–0.84)	1.00 0.75 (0.55–1.01)	1.00 0.55 (0.46–0.65)	7.67 (*P* = 0.0533)
Good health	No (ref) Yes	1.00 1.05 (0.97–1.13)	1.00 1.16 (0.93–1.45)	1.00 0.80 (0.56–1.13)	1.00 1.16 (0.95–1.40)	4.06 (*P* = 0.2547)
Has close friends	No (ref) Yes	1.00 0.80 (0.73–0.87)	1.00 0.51 (0.41–0.65)	1.00 0.52 (0.36–0.75)	1.00 0.57 (0.47–0.69)	21.29 (*P* < .0001)
Satisfied with life	No (ref) Yes	1.00 1.55 (1.46–1.64)	1.00 1.77 (1.47–2.14)	1.00 1.84 (1.32–2.55)	1.00 1.41 (1.18–1.69)	3.94 (*P* = 0.2685)
Can talk with parents	No (ref) Yes	1.00 0.50 (0.45–0.55)	1.00 0.42 (0.33–0.54)	1.00 0.35 (0.24–0.51)	1.00 0.32 (0.26–0.39)	18.52 (*P* = 0.0003)
Good financial situation	No (ref) Yes	1.00 1.09 (1.03–1.17)	1.00 1.06 (0.88–1.29)	1.00 1.01 (0.74–1.39)	1.00 0.88 (0.74–1.05)	5.23 (*P* = 0.1559)

Weekly sports activity was positively associated with weekly gambling among youth with Finnish-born parents, while the association with sports activity was negative among foreign-born youth. The statistically significant interaction showed that these groups differed with respect to this association (*P* = 0.0078).

Liking school, having close friends and communication with parents decreased the likelihood of weekly gambling in all origin groups but the interaction analysis showed that the effect of friends and parents was stronger among migrant origin youth. The effect of liking school was similar in all groups.

Satisfaction with life increased the likelihood of weekly gambling similarly in all groups while perception of good health was not associated with gambling in any of the groups.

Finally, poor financial circumstances were not associated with gambling in any of the migrant-origin groups, but good financial circumstances increased the probability of gambling among youth with Finnish-born parents.

## Discussion

The present study examined the association of origin with weekly gambling, and furthermore, the association of other known risk factors (substance use, sports activity, school orientation, general health, life satisfaction and peer and family related factors) with gambling behavior in different origin groups.

According to our findings, foreign-born, migrant-origin and youth from mixed families were more likely to gamble weekly compared to youth with Finnish-born parents. The likelihood of gambling was particularly high among foreign-born and migrant-origin youth. The association also remained statistically significant when other known risk and protective factors for gambling in adolescence were taken into account. The results are in line with those obtained by Canale et al. (2017), who found that problem gambling was twice as high among foreign-born compared with non-migrant youth. It has also been suggested that migrant-origin youth may experience greater adverse outcomes as a result of gambling, including a higher risk of taking up other addictive and risky behaviors due to acculturative stress, socio-economic circumstances, as well as access to and availability of gambling in permissive gambling cultures (Wardle et al., 2019). To continue, youth from mixed families may be subject to stress associated with an identity conflict. The integration of two identities and cultural backgrounds may be challenging, whereas bicultural identity can become a burden in adolescence, in a context where “normality” and peer identification are the strongest factors (Campisi et al., 2017).

Our study also confirmed previous findings related to gender differences in gambling (e.g., Buja et al., 2022; Claesdotter-Knutsson et al., 2022). Weekly gambling was significantly more common among boys than girls in all studied youth groups, but the association was stronger among youth with Finnish-born parents. Weekly gambling was particularly common among foreign-born boys compared with other groups.

Our results also showed that substance use was associated with weekly gambling and even more so among foreign-born youth. In addition, having close friends and being able to talk with parents had a stronger positive association with gambling among foreign-born youth compared to other peers. Weekly sports activity was positively associated with weekly gambling among youth with Finnish-origin parents, while the association with sports activity was negative among foreign-born youth.

A large number of studies confirm that perceived discrimination has negative effects on ethnic minority adolescents, such as depressive symptoms, suicide ideation, conduct problems, loneliness, lower levels of school engagement, and poorer academic achievement (Lemon et al., 2022; Priest et al., 2017; Ingram & Wallace, 2018; Galán et al., 2021; Assari et al., 2017; Lanier et al., 2016; Okoye & Saewyc, 2021). Perceived experiences of racism (individual, institutional or racial-ethnic microaggressions) can also lead to stress and coping challenges (Ingram & Wallace, 2018), which in turn can lead to addictive behaviors including gambling. Previous gambling studies have shown that adolescents currently experiencing difficulties with the acculturation process are more likely to experience gambling problems than those not currently experiencing such difficulties (Ellenbogen et al., 2007; Jacoby et al., 2013). Experiencing acculturative stress may lead to personal problems such as relationship difficulties with parents or in school, which may increase the likelihood of engaging in risky behaviors (Campisi et al., 2017), gambling being one example.

Adolescence is a time when youth begin to seek increased autonomy from parents, while social influences such as peers become more influential (Conger et al., 1991). During this developmental period, youth experience considerable social, emotional, physical, and cognitive changes that have implications for health and well–being over the course of life. Migrant-origin parents, on the other hand, may feel that their parenting ability is under serious stress in a new home-country and culture (e.g., Christie & Szorenyi, 2015). This stress may explain why among foreign-born youth, a good dialogical connection with the parents did not decrease the probability of weekly gambling, but rather the contrary. Stereotypical representation and negative qualities associated with migrant origin in dominant discourse and media may increase the likelihood of migrant-origin boys engaging in risky behaviors, such as gambling, and decrease their willingness to seek help.

Finally, contrary to our expectations, we did not find an association between poor financial situation and gambling in any of the studied groups. In fact, good financial circumstances increased the risk of gambling among youth with Finnish-born parents. Although financial problems in the family increase the risk of many kinds of problems in children’s health and wellbeing (Kääriälä et al., 2020), this does not seem to be the case regarding gambling, at least in our data on Finnish-origin adolescents. Some other non-financial motives seem to drive gambling behavior among foreign-born, migrant-origin, and youth from mixed families.

### Strengths and Limitations

This study filled an important gap in research by examining youth gambling behavior by origin, as well as factors associated with it with the SHP data. The dataset is unique in that it covers the whole cohort and is representative of 14 to 16-year-old Finnish schoolchildren. It is worth noting that the data collection in 2021 took place during the COVID-19 pandemic. It was not possible to consider the effects of the pandemic on youth gambling in our data. Very little is known about underage online gambling in Finland during the pandemic (Marionneau et al., 2022). However, there is no indication that Finnish adolescents would have gambled more online during the closures of electronic gambling machines placed outside the casinos.

As always with self-reporting data, there is a risk that students consciously or unconsciously don’t give accurate, honest answers about sensitive topics, such as gambling or substance use. The direction of these incorrect answers may go both ways, i.e., there may be over-reporting as well as under-reporting depending on what is socially desired/accepted in different contexts. However, a validity report on the European School Survey Project on Alcohol and other Drugs (ESPAD) shows that only a very small minority (1–2%) do not answer questions honestly that pertain to substance use. (Hibell et al., 2015). The data collection of the SHP used in this study is very similar to that of ESPAD, so there is a good reason to assume that these two surveys have captured youth gambling in a similar and reliable way and that the honesty of answering questions on gambling don’t differ from that of questions on substance use as these behaviors tend to cluster to same persons. As the data collection method has been similar in all included study years (2017, 2019 and 2021), the possible bias in responding has remained the same. Still, there is no reason to assume that the possible response bias to one direction or the other would affect the associations between the studied phenomena.

While a significant strength of the current study is the possibility to disaggregate the data by origin, as such information is rarely available in international studies on gambling among youth, we did not have data available on the specific region of origin or ethnicity of the participants. Thus, it is not possible to take the country of origin-specific habits and attitudes related to gambling into account, which is a limitation as differences between countries with regard to gambling certainly exist (ESPAD Group, 2020). It is also possible that limited knowledge of the Finnish language has caused difficulties in understanding and answering the questions among migrant-origin respondents. In this study, we also did not have data on religion, which may be a protective factor for gambling, especially if gambling is considered an immoral activity (Jacoby et al., 2013). To continue, the data didn’t include detailed information on socioeconomic position or contextual variables such as experiences of trauma, which might be associated with gambling. These limitations should be taken into account in future studies on risk behaviors among youth that focus on survey respondents’ immigrant status.

## Conclusions

Even though gambling has been forbidden for minors in Finland since 2011, a considerable proportion of youth still gamble. Although non-casino gambling nowadays requires identification, minors may gamble with someone else’s ID card, their gambling may be supported by older friends or relatives, or parents let their children play lottery games with them (Molinaro et al., 2018; Spångberg & Svensson, 2022). Age limits are an important tool to prevent underage gambling, but their implementation requires additional effort from the staff of non-casino premises. Youth gambling has not disappeared from Finland, and thus it is important that youth are continuously informed of the risks related to gambling. It is also important to continue informing youth of the risk related to substance use as part of efforts to reduce gambling, as these risky behaviors frequently go hand-in-hand.

Foreign-born boys appear to be especially vulnerable to multiple health and social risks including gambling, making them a particularly important group for targeted preventive programs. Gambling should not be seen as a positive pastime, as it is prohibited for minors due to the harms it may cause. To continue, it should not be seen as a tool to make money or to vent negative feelings. In addition, preventive efforts are needed to enhance public awareness, boost parental supervision, and limit gambling-related risks, and special attention is needed to prevent migrant-origin boys from developing problems with gambling.

## Data Availability

The data of this study are available from the Finnish Institute for Health and Welfare (THL), but restrictions apply to the availability of these data, which were used under licence for the current study and so are not publicly available. The data are, however, available from the authors upon reasonable request and with the permission of the Finnish Institute for Health and Welfare (THL).
